# The lncRNA NRON modulates HIV-1 replication in a NFAT-dependent manner and is differentially regulated by early and late viral proteins

**DOI:** 10.1038/srep08639

**Published:** 2015-03-02

**Authors:** Hasan Imam, Aalia Shahr Bano, Paresh Patel, Prasida Holla, Shahid Jameel

**Affiliations:** 1 Virology Group, International Centre for Genetic Engineering and Biotechnology, New Delhi, India

## Abstract

A majority of the human genome is transcribed into noncoding RNAs, of which the functions of long noncoding RNAs (lncRNAs) are poorly understood. Many host proteins and RNAs have been characterized for their roles in HIV/AIDS pathogenesis, but there is only one lncRNA, NEAT1, which is shown to affect the HIV-1 life cycle. We profiled 90 disease-related lncRNAs and found NRON (noncoding repressor of Nuclear Factor of Activated T cells [NFAT]) to be one of several lncRNAs whose expression was significantly altered following HIV-1 infection. The regulation of NRON expression during the HIV-1 life cycle was complex; its levels were reduced by the early viral accessory protein Nef and increased by the late protein Vpu. Consequently, Nef and Vpu also modulated activity of the transcription factor NFAT. The knockdown of NRON enhanced HIV-1 replication through increased activity of NFAT and the viral LTR. Using siRNA-mediated NFAT knockdown, we show the effects of NRON on HIV-1 replication to be mediated by NFAT, and the viral Nef and Vpu proteins to modulate NFAT activity through their effects on NRON. These findings add the lncRNA, NRON to the vast repertoire of host factors utilized by HIV for infection and persistence.

It is estimated that only 2% of the human genome codes for proteins, but over 70% of it is transcribed into RNA^1^. The protein-noncoding RNAs (ncRNAs) are divided into small ncRNAs (<200 nt) and long non-coding RNAs (lncRNAs) (>200 nt) based on their size^1^. The lncRNAs are important epigenetic regulators whose roles in disease processes are being increasingly recognized^2^. The possible mechanisms involved in lncRNA-mediated regulation include: translational modulation of mRNAs following sequence-specific recognition; targeting of chromatin modifiers to DNA through the formation of RNA-DNA hybrids; RNA secondary structure mediated targeting and sequestration of host factors; and as scaffolds to recruit multiple proteins into functional ribonucleoprotein complexes^3^. A recent annotation of lncRNAs produced by the human genome puts the number at 9277 genes and 14,880 transcripts^4^. While the small ncRNAs, especially the microRNAs (miRNAs) have been studied extensively for their roles in regulating gene expression during development and disease, studies on lncRNAs are limited. Although the pivotal role of individual lncRNAs in development and disease is being increasingly realized^5^, their possible roles in the pathogenesis of infectious disease have not received similar attention^6^.

The human immunodeficiency virus type 1 (HIV-1) contains nine genes that include the prototypic *gag, pol* and *env*, the regulatory *tat* and *rev*, and the accessory *nef, vif, vpr* and *vpu* genes^7^. For its replication, HIV-1 also utilizes a vast array of host factors^8^, while some host proteins such as APOBEC3G, BST2, etc., and miRNAs restrict viral replication^9^,^10^,^11^. To overcome intrinsic host restriction, HIV-1 accessory proteins such as Nef, Vif and Vpu play important and interesting roles^12^. Several reports also show the differential expression of host and viral miRNAs during HIV infection^13^,^14^,^15^, and host miRNAs to be predictive biomarkers of HIV/AIDS disease progression^16^. Despite rich literature on the interaction between HIV-1 and host factors, including miRNAs, there is only one published report on the role of endogenous lncRNAs in HIV-1 biology. Zhang et al profiled lncRNAs modulated in T-cell lines following HIV-1 infection, and characterized NEAT1 for its role in modulating post-transcriptional regulation of HIV-1 expression^17^.

To further study the relationship between HIV-1 and lncRNAs, we have profiled 90 disease-related lncRNAs in two human T-cell line models, and found several of these lncRNAs to be modulated following HIV-1 infection and replication. In this report, we show the lncRNA NRON to be downregulated in HIV-1 infected T cell lines and the viral accessory proteins Nef and Vpu to reciprocally regulate NRON levels. We further show that NRON modulates HIV-1 replication through its effects on the nuclear factor of activated T cells (NFAT).

## Results

### HIV-1 infection reduces NRON expression levels

**Figure 1:**
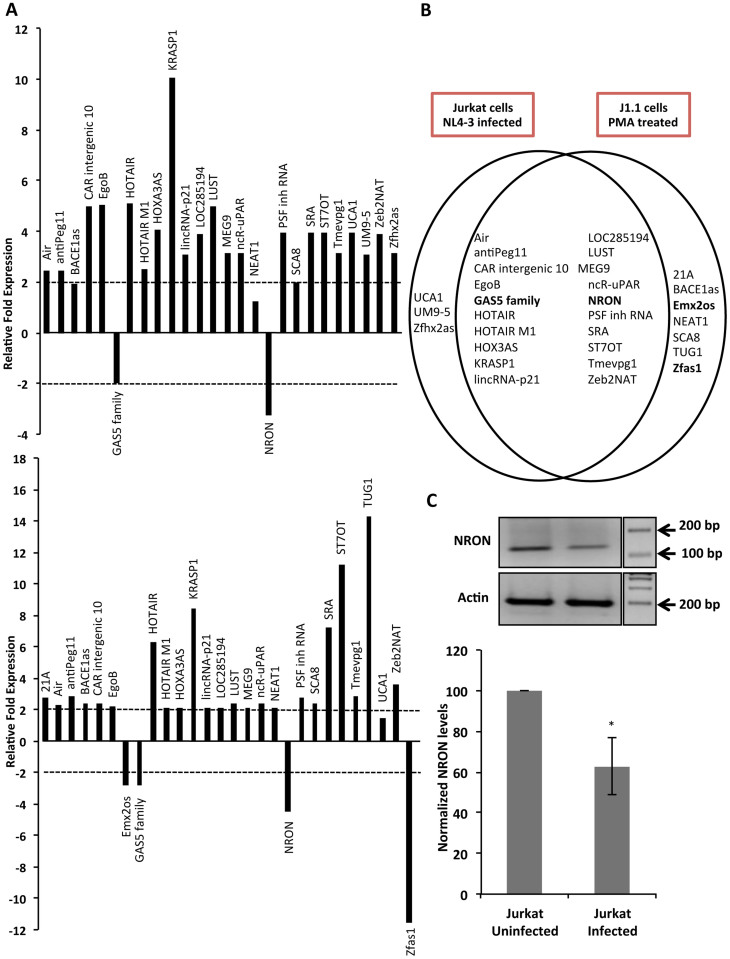
Differential expression of lncRNAs in HIV-1 infected cells. (A) Profiling results from Jurkat cells infected with HIV-1 NL4-3 (top) and J1.1 cells following PMA treatment (bottom). The plots show relative fold expression compared to control cells for three replicates. The dotted lines indicate the selected 2-fold cut-off. (B) Venn diagram summarizing the profiling results in the two experimental systems. The lncRNAs marked in bold were downregulated while others were upregulated. (C) Validation of NRON downregulation in HIV-1 NL4-3 infected Jurkat cells at 48 hpi by semi-quantitative RT-PCR. A representative cropped gel image is shown for NRON and Actin RNAs where the semi-quantitative RT-PCR products were run in a 2% agarose gel in same experimental condition. The full images are shown in Fig. S7A. The plot shows densitometry of gels from three separate experiments shown as mean values ± SD; * p<0.05.

To investigate if the expression of lncRNAs is altered by HIV-1 infection, we looked at 90 lncRNAs implicated in various diseases, including multiple cancers. Two cellular models were used, which included infection of the human Jurkat T-cell line with HIV-1, and J1.1 cells following PMA activation. The latter are Jurkat cells latently infected with HIV-1 and show robust viral replication and gene expression on activation with phorbol esters. From these two diverse yet related systems, we identified several lncRNAs that were modulated during HIV-1 infection and replication (Fig. 1A). The selection criteria included at least two-fold upregulation or downregulation, a p-value of < 0.05 and a qRT-PCR threshold cycle (C_T_) value of < 35. Based on these analyses, we found 2 lncRNAs (GAS5 family and NRON) to be downregulated and 21 lncRNAs to be upregulated in HIV-1-infected compared to mock-infected Jurkat cells. When compared to mock-activated cells, the PMA-activated J1.1 cells showed 4 lncRNAs (Emx2os, GAS5 family, NRON and Zfas1) to be downregulated and 23 lncRNAs to be upregulated. We found 2 downregulated and 18 upregulated lncRNAs to be common between the two cellular systems (Fig. 1B). An earlier study found the lncRNA NEAT1 to be upregulated in HIV infected Jurkat cells^17^. While we also found NEAT1 levels to increase, these were only 1.26-fold and 2.12-fold higher in HIV infected Jurkat cells and PMA activated J1.1 cells, respectively.

Among the identified lncRNAs, we pursued NRON because its expression was consistently downregulated the most in both experimental models. Further, NRON is a noncoding repressor of the transcription factor NFAT, which enhances HIV-1 gene expression in primary CD4 T cells^18^,^19^. We confirmed our profiling data by infecting Jurkat cells with HIV-1 NL4-3 at 1 moi for 48 hr and quantified NRON expression by semi-quantitative RT-PCR. Compared to mock-infected cells, HIV-1 infected Jurkat cells showed ~40% lower NRON levels (Fig 1C).

### The HIV-1 accessory proteins Nef and Vpu reciprocally regulate NRON levels and NFAT activity

**Figure 2:**
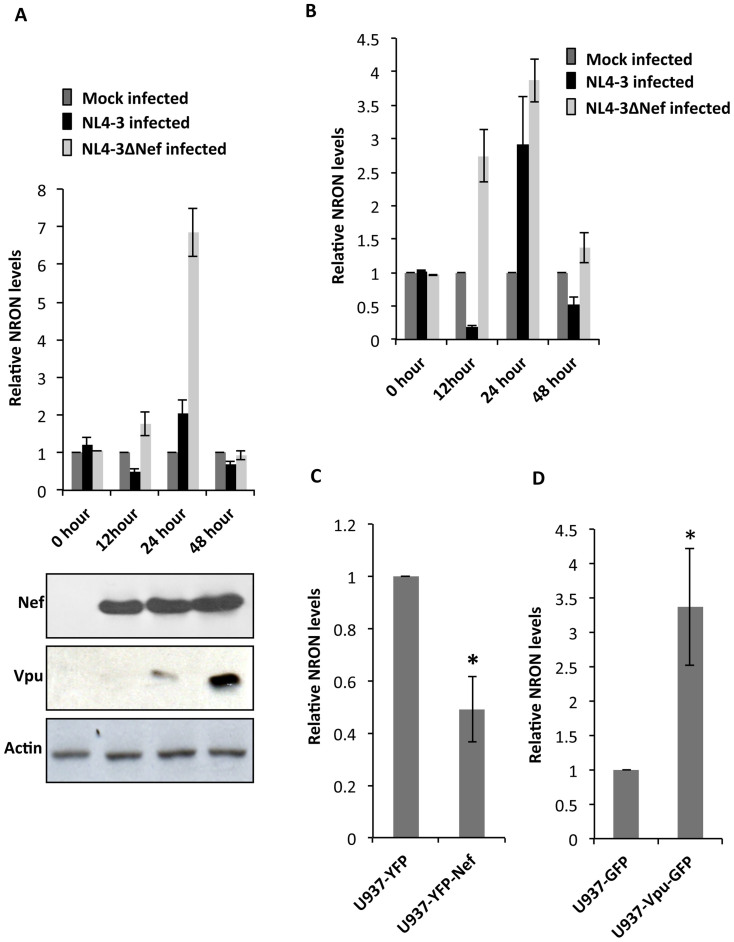
HIV-1 Nef and Vpu reciprocally regulate NRON expression. (A) Relative NRON expression levels at different times after infection of Jurkat cells with 1 moi of HIV-1 NL4-3 and HIV-1 NL4-3ΔNef viruses. The NRON levels were estimated by quantitative RT-PCR, were normalized to Actin and are expressed relative to levels found in mock-infected Jurkat cells. The cropped western blots show Nef and Vpu expression at different times after infection with HIV-1 NL4-3, with Actin as a loading control; the full images for these are shown in Fig. S7B. (B) Relative NRON expression levels at different times after infection of U937 cells with 1 moi of HIV-1 NL4-3 and HIV-1 NL4-3ΔNef viruses. The NRON levels were estimated by quantitative RT-PCR, were normalized to actin and are expressed relative to levels found in mock-infected U937 cells. (C) NRON expression levels in U937 cells stably expressing the Nef-EYFP fusion protein compared to cells stably expressing EYFP, at 48 hr after plating. (D) NRON expression levels in U937 stably expressing the Vpu-GFP fusion protein compared to cells stably expressing GFP, at 48 hr after plating. Results from three separate experiments are shown as mean values ± SD; * p<0.05.

We then carried out a kinetic assessment of the effects of HIV-1 infection on NRON in Jurkat cells and in U937 cells, which is a human monocytic cell line. We further tested whether the HIV-1 Nef accessory protein, which is expressed early during infection, might have a role in modulating NRON levels. Jurkat (Fig. 2A) or U937 (Fig. 2B) cells were infected with HIV-1 NL4-3 or a Nef-deficient variant, NL4-3ΔNef, and NRON levels were quantified at different times post-infection by qRT-PCR. At 12 hr post-infection (hpi), NRON levels go down in both cell types compared to mock-infected cells. Infection with Nef-deficient viruses did not show this decrease, but showed higher NRON levels. There were significant increases in NRON levels at 24 hpi, and these reduced substantially at 48 hpi. Nef was sufficiently expressed between 12 to 48 hpi, whereas the Vpu accessory protein started expressing at 24 hpi and attained good levels by 48 hpi. The results presented in Fig. 2A and 2B were normalized to mock-infected cells, which displayed stable NRON levels during this time period. Such complex regulation suggests that NRON levels might be regulated by more than one HIV-1 protein. Therefore, we used single-gene expression or knockout systems to tease out the effects of Nef and Vpu on NRON.

In U937 cells that stably expressed a Nef-EYFP fusion protein, we found reduced NRON levels compared to cells that expressed only EYFP (Fig. 2C). However, in U937 cells that stably expressed a Vpu-GFP fusion protein, the NRON levels were elevated (Fig. 2D). As earlier, U937 cells infected with HIV-1 NL4-3ΔNef also showed higher NRON levels than those infected with the full virus; there was reduced viral replication in cells infected with NL4-3ΔNef, as seen from p24^Gag^ levels (Fig. S1A). Analogous to this, infection of U937 (Fig. S1B) or Jurkat (Fig. S1C) cells with the NL4-3 and NL4-3ΔVpu viruses showed reduced NRON levels in the absence of Vpu. These results clearly show that while the Nef protein decreases NRON levels, the Vpu protein increased these.

**Figure 3:**
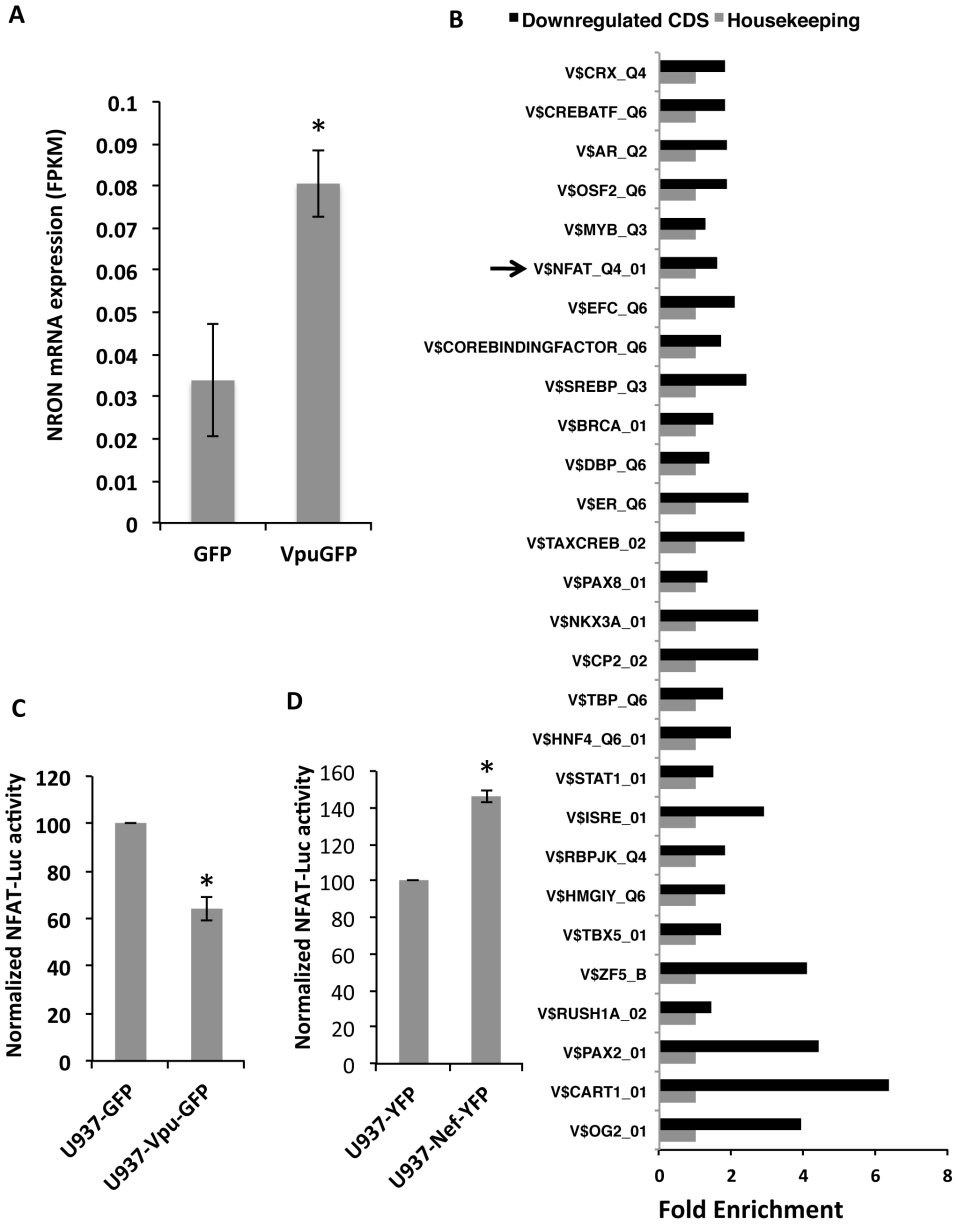
HIV-1 Nef and Vpu reciprocally modulate NFAT activity. (A) Expression levels of NRON RNA in U937 cells stably expressing Vpu-GFP compared to those in U937 cells stably expressing GFP deduced from RNA-Seq data. (B) Graphical representation of Match Analysis of NGS data as described in Methods. The transcription factor binding sites that are over-represented in downregulated coding sequences (CDS) are shown; arrow indicates NFAT. (C) Normalized NFAT-Luc activity in U937 cells stably expressing the Vpu-GFP fusion protein compared to cells expressing only GFP, at 48 hr after plating. (D) Normalized NFAT-Luc activity in U937 cells stably expressing the Nef-EYFP fusion protein compared to cells expressing only EYFP, at 48 hr after plating. Results from three separate experiments are shown as mean values ± SD. * p<0.05.

We then mined next generation sequencing (NGS) data available with us from U937/Vpu-GFP and U937/GFP cells (P. Patel et al; unpublished). The Integrated Genome Viewer (IGV) image (Fig. S2) showed sequence coverage in the NRON region, and differential expression of NRON was calculated using CuffDiff on three replicates. In agreement with qRT-PCR, the NGS results also showed increased expression of NRON in Vpu-expressing cells (Fig. 3A). Match Analysis of coding sequences provided lists of transcription factors whose sites were over-represented in either upregulated (not shown) or downregulated (Fig. 3B) coding sequences in Vpu-expressing cells. Interestingly, NFAT binding sites were over-represented in the upstream regions of genes that are downregulated in Vpu-expressing cells (Fig. 3B). To confirm this, we transfected U937/Vpu-GFP (or U937/GFP) and U937/Nef-EYFP (or U937/EYFP) stable cell lines with the NFAT-Luc reporter plasmid, and quantified luciferase activity in the cell lysates. There was reduced NFAT promoter activity in Vpu-expressing cells (Fig. 3C) and increased activity in Nef-expressing cells (Fig. 3D). Thus, analogous to their effects on NRON levels, Nef and Vpu reciprocally affect NFAT activity as well.

### NRON modulates HIV-1 replication through NFAT-mediated effects on the viral LTR

The experiments described above revealed the effects of HIV-1 infection and replication on cellular NRON levels. Since NRON represses the activity of NFAT, which is an important factor in activated T cells that promotes HIV-1 replication, it is also likely to influence the replication of HIV-1. To address this, we employed a short hairpin RNA (shRNA) to establish stable NRON knockdown lines in Jurkat cells. Control Jurkat cell lines were also established using a scrambled shRNA. Based on semi-quantitative RT-PCR (Fig. S3A) and qRT-PCR (Fig.S3B), the shNRON Jurkat cells showed 40–50% lower NRON levels compared to the scrambled shRNA control cells.

**Figure 4:**
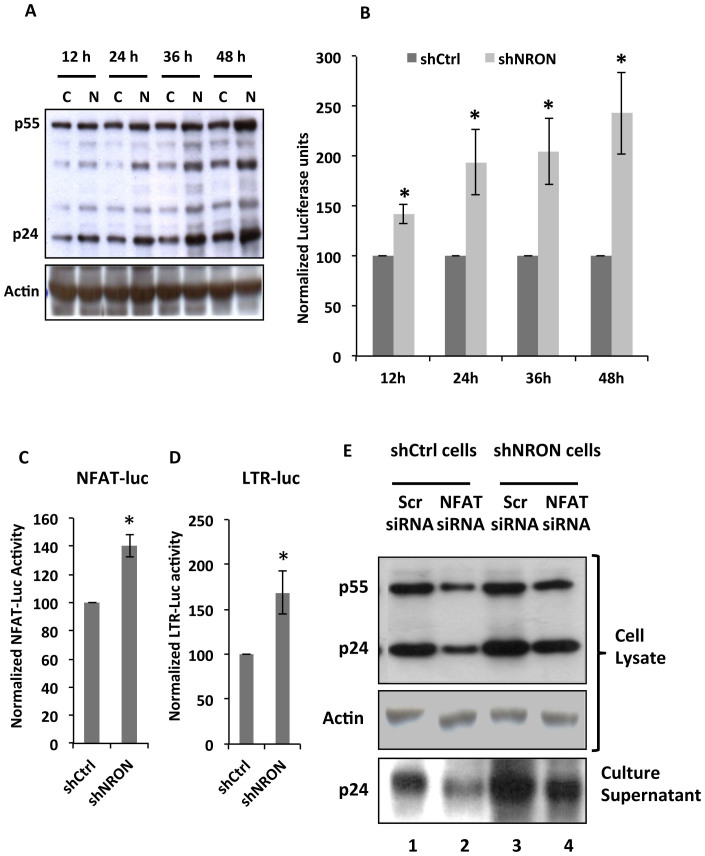
NRON modulates HIV-1 replication through NFAT-mediated LTR activation. (A) Control (lanes C) or NRON (lanes N) knockdown Jurkat cells were infected with 1 moi of HIV-1 NL4-3 and harvested at different times post-infection. Cropped western blot shows p55^Gag^ and p24^Gag^ in cell lysates, with Actin as a loading control; the full image is shown in Fig. S7C. (B) The graph shows results of TZMbl assay of corresponding supernatants of cells in (A). Data were normalized with the luciferase reading of control knockdown cells. (C) NFAT activity in NRON knockdown cells compared to control knockdown cells, estimated by transfection of pNFAT-luc reporter plasmid, at 48 hr post-transfection. (D) HIV-1 LTR activity in NRON knockdown cells compared to control knockdown cells, estimated by transfection of pHIV-luc reporter plasmid, at 48 hr post-transfection. The Firefly luciferase readings were divided by Renilla luciferase readings to normalize for transfection efficiency as described in Methods. (E) Effects on HIV-1 replication of NFAT knockdown in control and NRON knockdown cellular backgrounds. The NFAT siRNA transfections and analyses at 48 hr post-transfection were carried out as described in Methods. Cropped western blot (full image in Fig. S7D) shows p55^Gag^ and p24^Gag^ in cell lysates, and p24^Gag^ in supernatants. Actin served as a loading control for cell lysates. For quantitation, results from three separate experiments are shown as mean values ± SD; * p<0.05.

We then asked if HIV-1 replication rates were different in NRON knockdown and control Jurkat cells. For this, both cell lines were infected with 1 moi of HIV-1 NL4-3, and the cells and supernatants were harvested at 12, 24, 36 and 48 hpi. The cell lysates and supernatants were checked for Gag expression; equal volumes of the cell supernatants were also subjected to the TZMbl assay to estimate infectious virions released from the cells. At each time point, the NRON knockdown Jurkat cells produced more Gag (p55, p24 and intermediate forms) protein compared to the control cells (Fig. 4A). Importantly, at all time points the amounts of infectious virions produced per unit volume of culture supernatants were higher for NRON knockdown Jurkat cells compared to control cells (Fig. 4B).

The transcription factor NFAT is responsive to local changes in intracellular calcium and is crucial for the T-cell receptor-mediated immune response^20^. The stimulation of HEK293 cells with PMA/ionomycin, or shRNA-mediated knockdown of the NRON lncRNA was shown to result in increased NFAT activity^19^. We observed the same in Jurkat cells knocked down for NRON by transfecting the NFAT-luc reporter plasmid; these cells showed about 40% higher NFAT activity compared to control cells (Fig. 4C).

The HIV-1 LTR has multiple binding sites for lymphoid specific transcription factors and employs these for activation dependent viral gene expression^21^. NFAT is one such factor with two sets of binding sites identified in the HIV-1 LTR promoter that overlap the NFκB binding sites^18^. Since increased NFAT levels should also translate into increased HIV-1 LTR activity, we tested for this with transfection of an LTR-luc reporter plasmid. As expected, higher HIV-1 LTR activity was observed in NRON knockdown Jurkat cells compared to control cells (Fig. 4D).

To further confirm that NRON regulates HIV-1 replication through its effects on NFAT, we carried out siRNA-mediated knockdown of NFAT in the background of control or NRON knockdown Jurkat cells. To establish knockdown efficiency, we first transfected HEK293T cells with either an NFAT-specific siRNA or a control non-targeting siRNA, and followed this up by transfection of the NFAT-luc reporter plasmid. There was reduced NFAT activity in cells that received the NFAT siRNA compared to the control siRNA (Fig. S3C). This was then repeated in the control or NRON knockdown Jurkat cell lines with the same results (Fig. S3D). Finally, we evaluated HIV-1 replication in these cells by western blotting for p55^Gag^ and p24^CA^ in cell lysates and for new virions (p24^CA^) in culture supernatants. Cells knocked down for NRON showed increased Gag expression (Fig. 4E; lanes 1 and 3), as observed earlier (Fig. 4A), and is due to higher levels of NFAT. Cells receiving the NFAT siRNA showed reduced Gag expression (Fig. 4E; lanes 1 and 2) since functional NFAT is reduced in these cells through the siRNA as well as NRON. However, NRON knockdown cells that received the NFAT siRNA showed relatively higher levels of Gag (Fig. 4E; lanes 2 and 4) because functional NFAT in these cells is reduced only through the siRNA. There was a severe reduction in intracellular Gag levels and secreted virus on NFAT knockdown, but this recovered significantly when the NFAT reduction was in the NRON knockdown background (Fig. S4A). This was also true for gag RNA (Fig. S4B).

The lncRNA NRON binds to NFAT and inhibits its nuclear translocation. We activated control or NRON knockdown cells with PMA/Ionomycin and tested for NFAT levels in whole cell lysates, nuclear and cytoplasmic fractions by western blotting (Fig. S5). We also stained cells for NFAT and quantified the signals and its colocalization with the nuclear marker DAPI (Fig. S6). NFAT levels were higher in NRON knockdown cells compared to control cells (Fig. S5A). This was also the case with nuclear levels of NFAT, which increased further on activation (Fig. S5B). Fluorescent imaging and quantification of NFAT (Fig. S6) agreed with the fractionation and western blotting results, showing increased nuclear translocation of NFAT in NRON knockdown cells. Together, these results support our hypothesis that the modulation of HIV-1 replication by NRON is mediated through NFAT.

### The effects of HIV-1 on NFAT activity are directed through NRON

**Figure 5:**
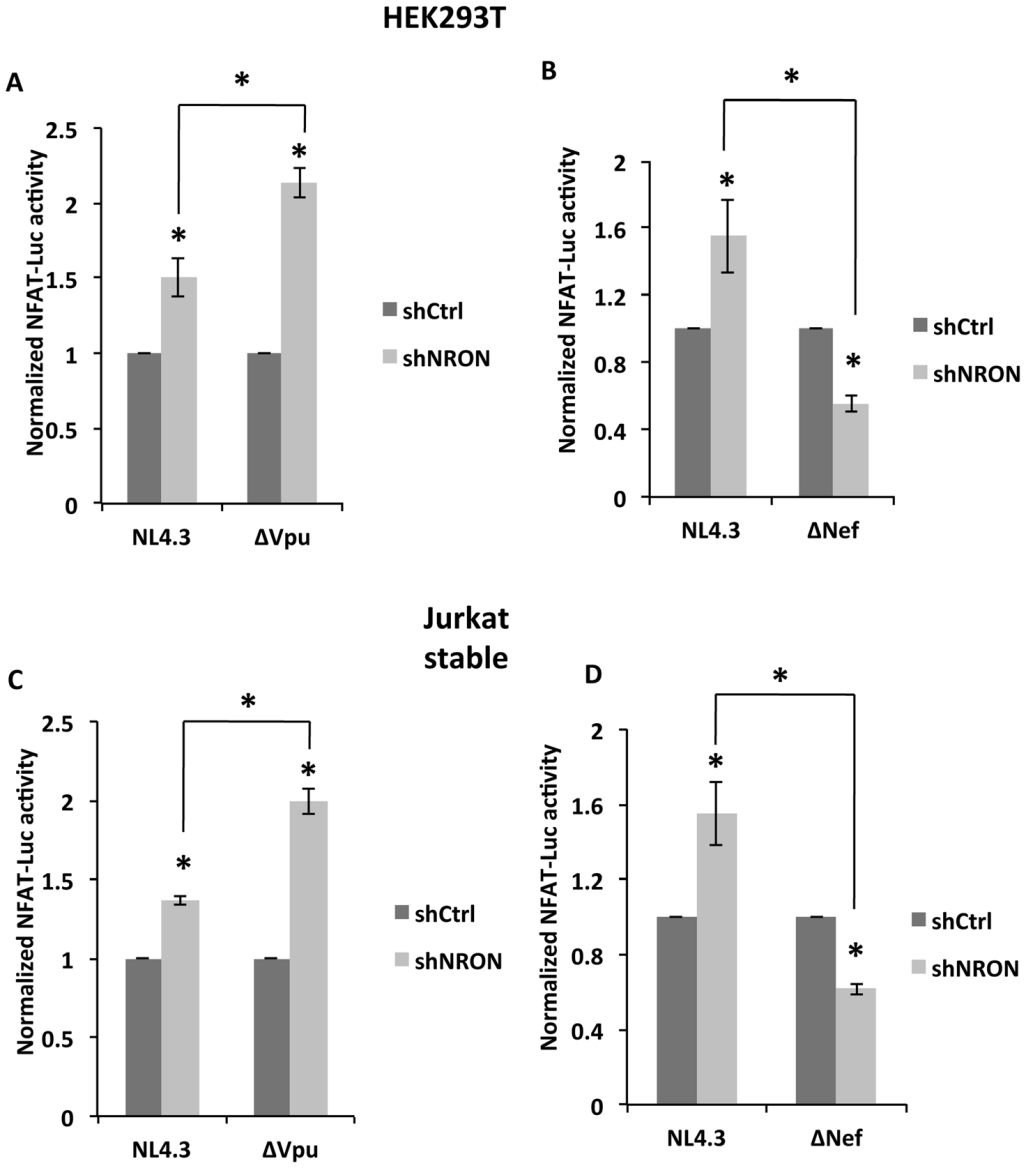
HIV-1 Nef and Vpu alter NFAT activity through their effects on NRON. (A) NFAT activity in (A) HEK293T cells transfected with plasmids pNL4-3 or pNL4-3ΔVpu together with either control shRNA or NRON shRNA plasmids, and the NFAT-luc reporter plasmid; (B) HEK293T cells transfected with plasmids pNL4-3 or pNL4-3ΔNef together with either control shRNA or NRON shRNA plasmids, and the NFAT-luc reporter plasmid; (C) Control or NRON knockdown Jurkat cells transfected with plasmids pNL4-3 or pNL4-3ΔVpu and the NFAT-luc reporter plasmid; and (D) Control or NRON knockdown Jurkat cells transfected with plasmids pNL4-3 or pNL4-3ΔNef and the NFAT-luc reporter plasmid. Transfections and luciferase estimations were carried out as described in Methods. All measurements were made at 48 hr post-transfection. Results from three separate experiments are shown as mean values ± SD; * p<0.05.

Previously we showed that Nef decreased NRON levels and Vpu increased these. Further, these HIV-1 proteins reciprocally altered NFAT activity as well, with Nef increasing it and Vpu decreasing it. To prove that the NFAT activity changes were due to varying NRON levels, we first co-transfected HEK293T cells with the infectious HIV-1 plasmids pNL4-3, pNL4-3ΔVpu or pNL4-3ΔNef, together with the shNRON (or control shRNA) plasmid and the NFAT-luc reporter plasmid. The NFAT activity was always higher in the NRON knockdown background when wild type or Vpu-deficient HIV-1 was present, but was lower in the case of Nef-deficient HIV-1 (Fig. 5A, B). The same experiment was then repeated in Jurkat cells that were stably knocked down for NRON. In this case, one of the three infectious HIV-1 plasmids was nucleofected in NRON knockdown (or control) Jurkat cells together with the NFAT-luc reporter plasmid. Again, NFAT activity was higher in the NRON knockdown background, except when Nef-deficient HIV-1 was used (Fig. 5C, D). These results show that during viral infection, the Nef and Vpu proteins regulate NFAT activity by modulating NRON expression.

## Discussion

We report here only the second example in literature for lncRNA involvement in HIV-1 replication. Our profiling studies showed HIV-1 infection to significantly reduce the intracellular levels of NRON in two human T-cell line models of infection. Interestingly, the HIV-1 accessory Nef protein, which is expressed early in the viral life cycle reduced NRON levels, but the accessory Vpu protein expressed late in replication, increased NRON levels. Jurkat cells stably knocked down for NRON expression also showed increased HIV-1 replication.

On comparing our study with that of Zhang et al^17^, we note that NRON was not part of their lncRNA array. Like that study, we also observed NEAT1 to be upregulated in HIV-1 infected cell models, but the increases had borderline significance. A close scrutiny showed the GAS5 family lncRNAs to be downregulated in both studies and the following lncRNAs to be upregulated: Air, antiPeg11, CAR intergenic 10, EgoB, HOTAIR, HOTAIR M1, HOX3AS, KRASP1, lincRNA-p21, LOC285194, LUST, MEG9, ncR-uPAR, PSF inh RNA, SRA, ST7OT, Tmevpg1 and Zeb2NAT. Some common themes emerge from the known functions of these lncRNAs^22^ that might be of relevance to HIV-1 infection and persistence. The GAS5 family, KRASP1, lincRNA-p21 and LUST lncRNAs are responsible for reduced growth arrest and apoptosis, and increased cell proliferation, which positively impacts HIV-1 infection and replication. At least four lncRNAs – Air, HOTAIR, antiPeg11 and MEG9 are involved in epigenetic silencing, of which HOTAIR and antiPeg11 bind and recruit the polycomb repressor complex 2 (PRC2). It was shown earlier that PRC2-mediated silencing is important for HIV-1 latency^23^. The lncRNA Tmevpg1 is expressed in NK, CD4+ and CD8+ cells, and cross-regulates gamma interferon^24^, which is an important proinflammatory cytokine during HIV infection. Interestingly, HIV also encodes an antisense lncRNA that regulates viral transcription by altering the epigenetic landscape at the viral promoter^25^.

The lncRNA NRON is about 2.7 kb in length and is composed of three exons, which can be alternatively spliced to yield transcripts ranging in size from 0.8 to 3.7 kb^19^. It was identified as a noncoding RNA repressor of the transcription factor NFAT, which regulated the nuclear trafficking of NFAT^19^. The NFAT family has five members, NFAT1 to NFAT5, of which all except NFAT5 are calcium regulated transcription factors, and NFAT1 is the predominant family member expressed in primary human CD4 T cells^26^. In resting T cells, NFATs are phosphorylated and retained in cytoplasm by the NRON complex^27^. Following calcium stimulation, NFAT proteins are dephosphorylated by the Ca^2+^/calmodulin-dependent phosphatase, Calcineurin, and translocate to the nucleus to activate gene expression^28^. The NFAT proteins bind the NFκB sites in the HIV-1 LTR, and NFAT1 and NFAT2 were shown to increase HIV-1 transcription and replication in primary human CD4 T cells and Jurkat cells^29^,^30^. As a non-coding repressor of NFAT, the lncRNA NRON may also modulate HIV transcription and replication. Indeed, our results show that knockdown of NRON in Jurkat cells leads to increased NFAT activity, HIV-1 LTR activity and HIV-1 replication.

The Nef protein is expressed very early following HIV-1 infection, and is reported to have multiple activities that promote viral replication. This includes its ability to coordinate T cell activation through synergistic activation of the calcium/calcineurin and Ras-Raf-MAPK signaling pathways and induction of NFAT^31^. In the absence of further stimulation, HIV replication in CD4+ T lymphocytes is supported by the overexpression of NFAT target genes like IL-2 and FasL^32^. Additionally, NFAT proteins interact with the NF-κB responsive element and activate HIV-1 LTR directed transcription^32^. We confirmed these effects of Nef on NFAT and showed these to be mediated by NRON expression. We also made the novel observation that Vpu, which is expressed late in HIV-1 infection, decreases NFAT activity by increasing NRON levels. Thus, NRON appears to act as a rheostat that finely tunes the degree of T-cell activation and HIV-1 LTR mediated transcription by controlling NFAT activity. This is likely to be important for maintaining a balance between viral replication and activation-mediated T-cell death.

**Figure 6:**
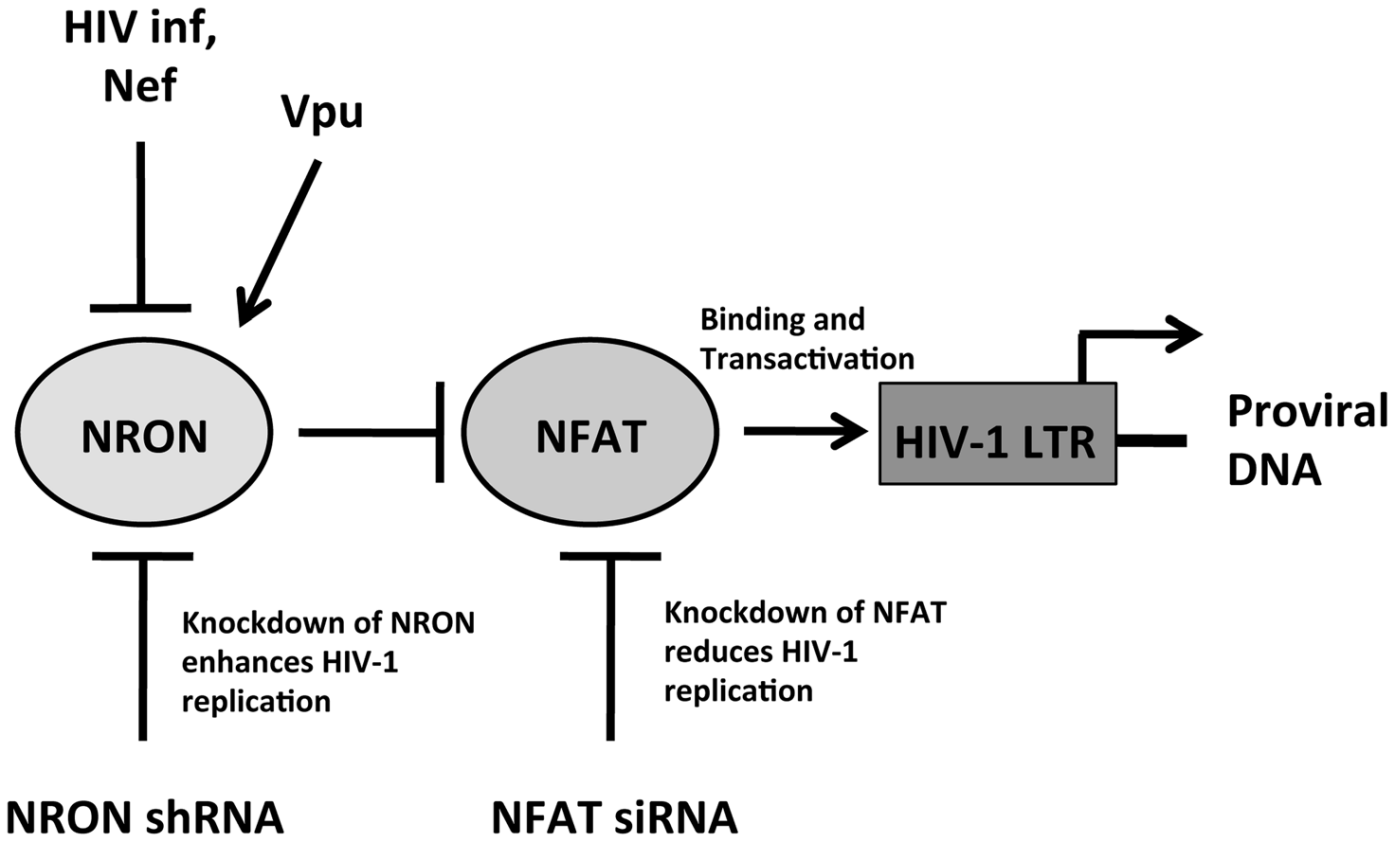
Proposed model for cross-regulation of NRON and HIV-1 replication. The model shows HIV-1 infection and the viral Nef protein to reduce NRON levels, and the viral Vpu protein to increase NRON levels. This in turn regulates activity of the transcription factor NFAT, which enhances transcription from the HIV-1 LTR. The interventions with shRNA-mediated NRON knockdown and siRNA-mediated NFAT reduction, are shown.

Our approach and findings are summarized in the model shown in Figure 6. The replication of HIV-1 in CD4+ T cells or the expression of Nef results in reduced NRON levels, leading to NFAT activation and increased transcription from the HIV-1 LTR. This, together with NFAT-mediated T-cell activation, has an overall positive effect on HIV-1 replication. Later in the viral life cycle, the late accessory Vpu protein increases NRON levels, possibly attenuating T-cell activation and death. Exactly how Nef and Vpu modulate NRON expression levels remains an interesting question to be addressed in future studies.

## Methods

### Plasmids and antibodies

The pNL4-3, pNL4-3ΔNef and pNL4-3ΔVpu infectious molecular clones of HIV-1 subtype B were obtained from the NIH AIDS Research and Reagent Program (NIAID, Rockville, MD, USA). The NFAT-Luc (#17870) reporter plasmid was obtained from Addgene (deposited by Dr. Gerry Crabtree), and the LTR-Luc reporter plasmid was from Dr. Akhil Banerjea, National Institute of Immunology, New Delhi, India. The scrambled shRNA and NRON-specific shRNA sequences were cloned in the pSIREN-Retro Q-Zs green1 vector^33^. The NRON shRNA sequence^19^ was as follows: Forward oligo 5′-GATCCGCTGTTTCCACTACTGCTCCTTCAAGAGAGGAGCAGTAGTGGAAACAG TTTTTTCTAGAG-3′, Reverse oligo 5′-AATTCTCTAGAAAAAAACTGTTTCCACTACT GCTCCTCTCTTGAAGGAGCAGTAGTGGAAACAGCG-3′. Anti-p24 (hybridoma sup) was from the NIH AIDS Research and Reagent Program (NIAID, Rockville, MD, USA), anti-Histone, anti-GAPDH and anti-Actin antibodies were from Santa Cruz Biotechnology (USA), anti-NFAT antibody was from Abcam (UK), anti-mouse IgG-HRP was from Calbiochem (USA) and Alexa Fluor488 and Alexa Fluor594 conjugated secondary antibodies was from Molecular Probes (Invitrogen, USA).

### LncRNA profiling

HIV-1 was produced in HEK293T cells transfected with plasmid pNL4-3; the culture supernatants were harvested 48 hr post-transfection and infectious titers were estimated on TZMbl cells^34^. For lncRNA profiling, Jurkat cells were infected with HIV-1 at 1.0 moi and J1.1 cells were activated with 50 ng/ml phorbol 12-myristate 13-actetate (PMA). Two days later, culture supernatants and cells were harvested and western blotting for p24 was used to check viral replication. Total RNA was extracted from cells using Trizol reagent (Invitrogen), following the manufacturer's instructions. The same amounts of RNA were converted to cDNA and the Human LncRNA Profiler Array kit (System Biosciences) was used to profile lncRNAs in the samples according to the manufacturer's instructions. The 90 lncRNAs profiled were as follows: 21A, 7SK, 7SL, Air, AK023948, Alpha 280, Alpha 250, ANRIL, anti-NOS2A, antiPeg11, BACE1AS (family), BC200, CAR Intergenic 10, DHFR upstream transcripts (family), Dio3os (family), DISC2 (family), DLG2AS (family), E2F4 antisense, EgoA, EgoB, Emx2os, Evf1 and EVF2, GAS5-family, Gomafu, H19, H19 antisense, H19 upstream conserved 1 & 2, HAR1A, HAR1B, HOTAIR, HOTAIRM1, HOTTIP, Hoxa11as, HOXA3as, HOXA6as, HULC, IGF2AS (family), IPW, Jpx, Kcnq1ot1, KRASP1, L1PA16, lincRNA-p21, lncRNA-RoR, lincRNA-SFMBT2, lincRNA-VLDLR, LOC285194, LUST, Malat1, mascRNA, MEG3 (family), MEG9, MER11C, ncR-uPAR, NDM29, NEAT1 (family), Nespas, NRON, NTT, p53 mRNA, PCGEM1, PR antisense transcripts, PRINS, PSF inhibiting RNA, PTENP1, RNCR3, SAF, SCA8, snaR, SNHG1, SNHG3, SNHG4, SNHG5, SNHG6, Sox2ot, SRA, ST7OT, TEA ncRNAs (family), Tmevpg1, TncRNA, Tsix, TUG1 (family), UCA1, UM9-5, WT1-AS, Xist, Y RNA-1, Zeb2NAT, Zfas1 and Zfhx2as. The MIHS excel format (Qiagen, Germany) was used for analysis of the PCR array data. In this analysis, Ct values >35 were automatically excluded and four endogenous controls (GAPDH, 18S rRNA, U6 snRNA and RNU43) were considered in the analysis. The output included fold changes in the corresponding lncRNAs in HIV-1 infected or PMA activated cells with respect to the control cells, and the p values for these changes.

### Cell culture, transfection and infection

The stable cell lines U937/Vpu-GFP, U937/GFP, U937/Nef-EYFP and U937/EYFP have been described elsewhere^35^,^36^. These cells as well as Jurkat and J1.1 cells^37^ (obtained from NIH AIDS Research and Reagent Program; NIAID, Rockville, MD, USA) were maintained in RPMI 1640 supplemented with 10% fetal bovine serum (FBS). The TZMbl and HEK293T cells were maintained in Dulbecco's modified Eagle's medium (DMEM) with 10% FBS. Plasmids were transfected into HEK293T cells using jetPRIME reagent (Polyplus Transfection Co., USA). For transfection into Jurkat cells, 2 million cells were suspended in 100 µl of Solution V (Nucleofector kit V; Amaxa Biosystems) and mixed with 4 µg plasmids or 50 nM siRNAs, and processed following the manufacturer's instructions. The Nef- and Vpu-defective viruses were produced and tittered as described above for HIV-1. All infections were carried out at 1.0 moi.

### RNA inhibition

To prepare shRNA stable lines, plasmids containing the NRON or control shRNA (6 µg) were transfected into 2 million HEK293T cells along with plasmids pVSV-g (1.5 µg) and pGag-Pol (3 µg). The culture supernatants were collected after 48 hr, filtered through 0.45 µm filters and 10 ml was used to directly infect 5 million Jurkat cells. After 48 hr, cells expressing GFP were sorted out at the Stem Cell Biology Lab, National Institute of Immunology (New Delhi, India) and thereafter maintained in RPMI 1640 containing 10% FBS. For siRNA-mediated knockdown of NFAT, the ON-TARGET plus Human NFATC1 siRNA SMART pool (4772) (Thermo Fisher Scientific, Lafayette, USA) was used; the ON-TARGET plus Non-targeting siRNA#1 (D-001810-01-20) was used as the scrambled control.

### NGS and Match Analysis

The RNA from U937/Vpu-GFP (and control U937/GFP) stable cell lines was sequenced at the Center for Cellular and Molecular Platforms (c-CAMP), Bengaluru, India. The paired end RNA-seq was performed on Illumina-HiSeq 1000 with the insert size of around 140-200 bases. The kits used for sample preparation were TruSeq RNA sample preparation kit RS-122-2001 and RS-122-2002. For Cluster generation, TruSeq PE Cluster Kit V3, and for sequencing the FC-401-3001-TruSeq SBS Kit V3-HS were used. Differential expression analysis was performed using TopHat (http://ccb.jhu.edu/software/tophat/index.shtml) and Cufflinks packages at the default settings (http://cufflinks.cbcb.umd.edu/). The MATCH program (http://www.biobase-international.com/product/explain) was used to scan the −500 to +100 nucleotides in the promoter regions of differentially regulated coding sequences (CDS) using collections of known transcription factor binding site (TFBS) and positional weight matrices, to identify binding sites for a given transcription factor in the upregulated or downregulated CDS. Promoters from human housekeeping genes were taken as a control set.

### Semi-quantitative and quantitative PCR

The miScript II RT kit (Qiagen, Germany) was used to prepare cDNA and quantitative PCR was done with SYBR green master mix (Solis BioDyne, Estonia) using the following primers: NRON forward, 5′-ACGTTCCTTAATGTACGCCTTTGC-3′, NRON reverse, 5′-TTGGCCGTGTCCTGAGTC CTT-3′; Actin forward, 5′-TGGCGCTTTTGACTCAGGAT-3′, Actin reverse, 5′-GTTACT ACCCAGGTCAGGCCAG-3′; Gag forward, 5′-ACCCATGTTTCAGCATTAT-3′, Gag reverse 5′-GCTTGATGTCCCCCTACTGT-3′. The qRT-PCR program was 95°C for 10 min followed by 40 cycles at 95°C for 30 sec, 60°C for 30 sec, 72°C for 30 sec, and fold changes in gene expression were calculated by the ΔΔCT method. For semi-quantitative PCR, the program was 95°C for 1 min followed by 25 cycles at 95°C for 30 sec, 60°C for 30 sec and 72°C for 30 sec, and fold changes in gene expression were calculated based on densitometry using the NIH Image J software to determine band intensities.

### Western blotting

Cell pellets were washed twice with ice-cold PBS and lysed with 1X SDS protein loading buffer (50 mM Tris, 2% SDS, 10% glycerol, 2% β-mercaptoethanol, and 0.1% bromophenol blue). The samples were boiled at 95°C for 5 min and clarified lysates were resolved by SDS-10%-PAGE and transferred to polyvinylidene fluoride (PVDF) membranes (MDI, Karnal, India). The membrane was blocked with 5% BSA for 1 hour, followed by overnight incubation with primary antibodies (1:1000) diluted in 2% BSA. After washing, the membranes were incubated with HRP-conjugated secondary antibodies (1:5000) for 1 hour. The signals were detected using a chemiluminescence substrate (Santa Cruz Biotechnology, USA). The NIH Image J software was used for blot densitometry.

### TZMbl assay

The TZMbl cells were seeded in the wells of a 96-well plate (10,000 cells/well) with complete DMEM. On the next day, medium was removed from each well and cells were washed once with 200 µl incomplete DMEM. Cells were starved for 1 hr in 100 µl incomplete DMEM, which was then removed and different dilutions of virus were added in a total volume of 100 μl of incomplete DMEM. After 4–6 hr of incubation at 37°C in a 5% CO_2_ incubator, the virus containing medium was removed and 100 µl of complete medium was added. After 48 hr of incubation, the luciferase reading was taken using Steady-Glo luciferase assay reagent according to manufacturer's instructions (Promega, Madison, USA).

### Luciferase assay

Cells were transfected with the Firefly luciferase reporters as described above together with plasmid pRLTK that constitutively expresses Renilla luciferase. Cell lysates were assayed for Firefly and Renilla luciferase activities using the Dual Luciferase Assay System (Promega, Madison, USA) as per manufacturer's instructions. All Firefly luciferase readings were divided by Renilla luciferase readings to normalize for transfection efficiency of cells.

### Cell fractionation

Control and NRON knockdown cells were stimulated for 3 hr with 5 ng/ml phorbol 12-myristate 13-acetate (PMA, Sigma) and 1.5 ug/ml Ionomycin (Sigma). For preparation of cytoplasmic and nuclear fractions, the harvested cells were lysed in Triton X-100 lysis buffer (50 mM Tris HCl (pH 7.5), 0.5% Triton X-100, 137.5 mM NaCl, 10% glycerol, 1 mM sodium vanadate, 50 mM sodium fluoride, 10 mM sodium pyrophosphate, 5 mM EDTA and protease inhibitor cocktail) on ice for 15 min. The mixture was then centrifuged at 3000 rpm for 5 min at 4°C in a microfuge. The supernatant was saved as the cytoplasmic fraction. The pellet was resuspended in equal volume of Triton X-100 lysis buffer and centrifuged at 13000 rpm for 15 min at 4°C in a microfuge; the supernatant served as the nuclear fraction. The protein levels were estimated using the Bradford assay (BioRad) and equal amounts of total proteins were western blotted. Histone H1 and GAPDH served as nuclear and cytoplasmic markers, respectively.

### NFAT microscopy and quantification

For immunofluorescence staining, control and NRON knockdown stable Jurkat cells were activated with PMA/Ionomycin for 6 hr, washed, fixed with 2% paraformaldehyde for 10 min and treated with the permeabilization buffer (Perkin Elmer, USA). Cells were then blocked with 5% BSA, incubated with anti-NFAT antibody (1:500 in 1% BSA solution) for 1 hr, followed by Alexa Fluor594 conjugated anti-mouse secondary antibody (1:1500 in 1% BSA solution) for 1 hr. Cells were then mounted on glass slides with Prolong Gold DAPI+antifade solution (Life Technologies, USA) and visualized on a Nikon A1R laser scanning confocal microscope. Fluorescence intensity measurements and image processing were carried out using NIS Elements microscope imaging software.

### Statistical analyses

Analyses of the lncRNA, NGS, quantitative and semi-qunatitative PCR data were carried out as detailed in the respective sections. Statistical significance was calculated by paired two-tailed T Test and p<0.05 was considered significant.

## Author Contributions

H.I., A.S.B., P.P., P.H. and S.J. designed the experiments and analyzed the data; H.I., A.S.B., P.P. and P.H. performed the experiments; H.I. and S.J. wrote the manuscript; S.J. supervised the project.

## Supplementary Material

Supplementary Information Supplementary Figures
