# Supplementary material for: The lncRNA NRON modulates HIV-1 replication in a NFAT-dependent manner and is differentially regulated by early and late viral proteins
Source: Sci Rep. 2015 Mar 2;5:8639. doi: 10.1038/srep08639 (PMC4345339; doi:10.1038/srep08639)
Supplement: Supplementary Information — Supplementary Figures [file srep08639-s1.doc]

**The lncRNA NRON modulates HIV-1 replication in a NFAT-dependent manner and is differentially regulated by early and late viral proteins**

**Hasan Imam, Aalia Shahr Bano, Paresh Patel, Prasida Holla and Shahid Jameel**

**
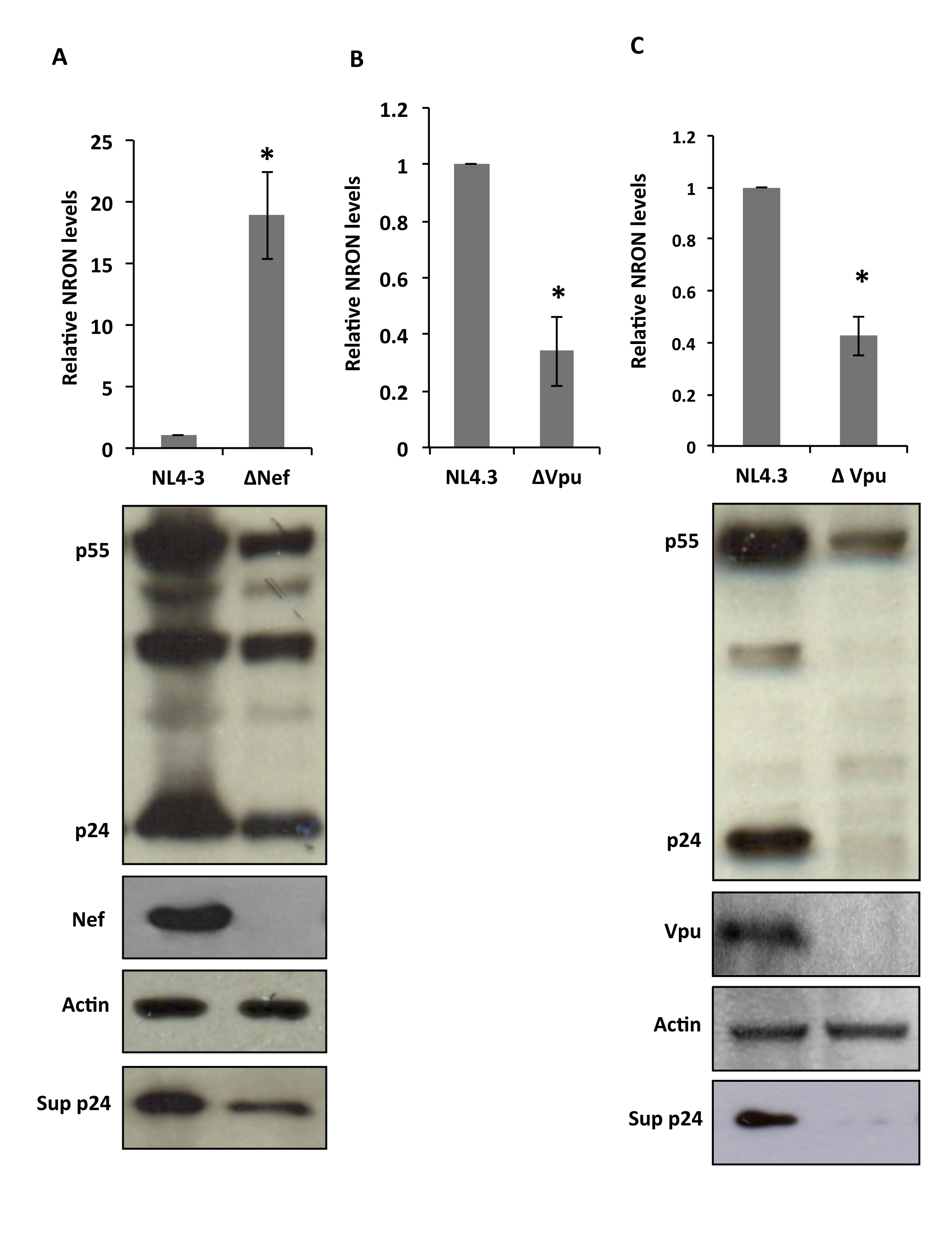
**

**Figure S1: Nef and Vpu reciprocally modulate NRON levels.** (A) Relative NRON expression levels in U937 cells after infection with 1 moi of either HIV-1 NL4-3 or NL4-3∆Nef viruses, at 48 hpi. NRON levels were estimated by quantitative RT-PCR. Cropped western blot figures show corresponding p55^Gag^, p24^Gag^and Nef levels in cell lysates and p24^Gag^ levels in culture supernatants; Actin served as a loading control. (B) Relative NRON expression levels in U937 cells after infection with 1 moi of either HIV-1 NL4-3 or NL4-3∆Vpu viruses, at 48 hpi. (C) Relative NRON expression levels in Jurkat cells after infection with 1 moi of either HIV-1 NL4-3 or NL4-3∆Vpu viruses at 48 hpi. NRON levels were estimated by quantitative RT-PCR. The quantitative NRON results from three separate experiments are shown as mean values +SD; * p<0.05. Cropped western blot figures show corresponding p55^Gag^, p24^Gag^ and Vpu levels in cell lysates and p24^Gag^ levels in culture supernatants; Actin served as a loading control. The western blots are representative.


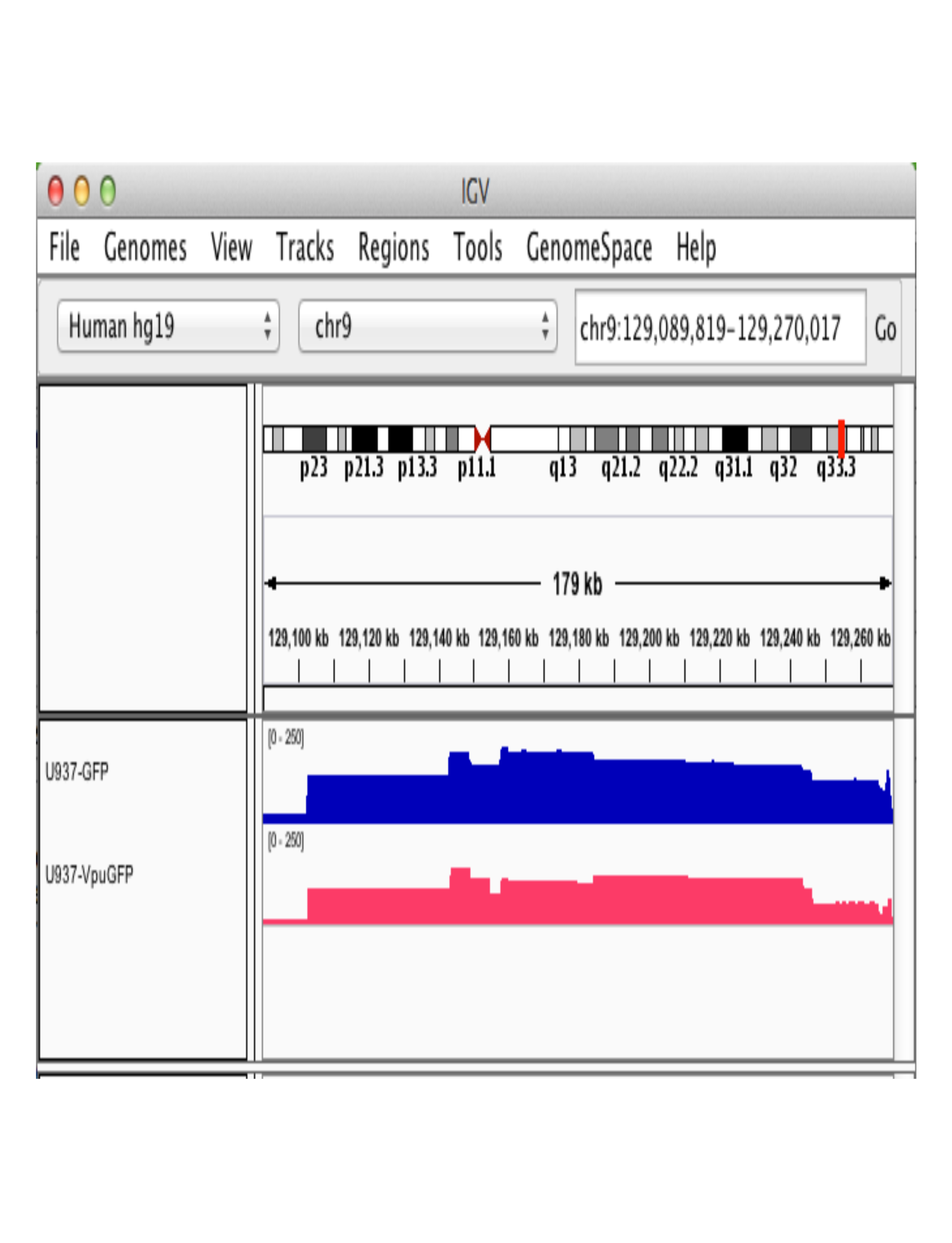


**Figure S2:** Integrated Genome Viewer (IGV) image showing NRON region coverage of U937-GFP and U937-VpuGFP reads from one representative set.

**
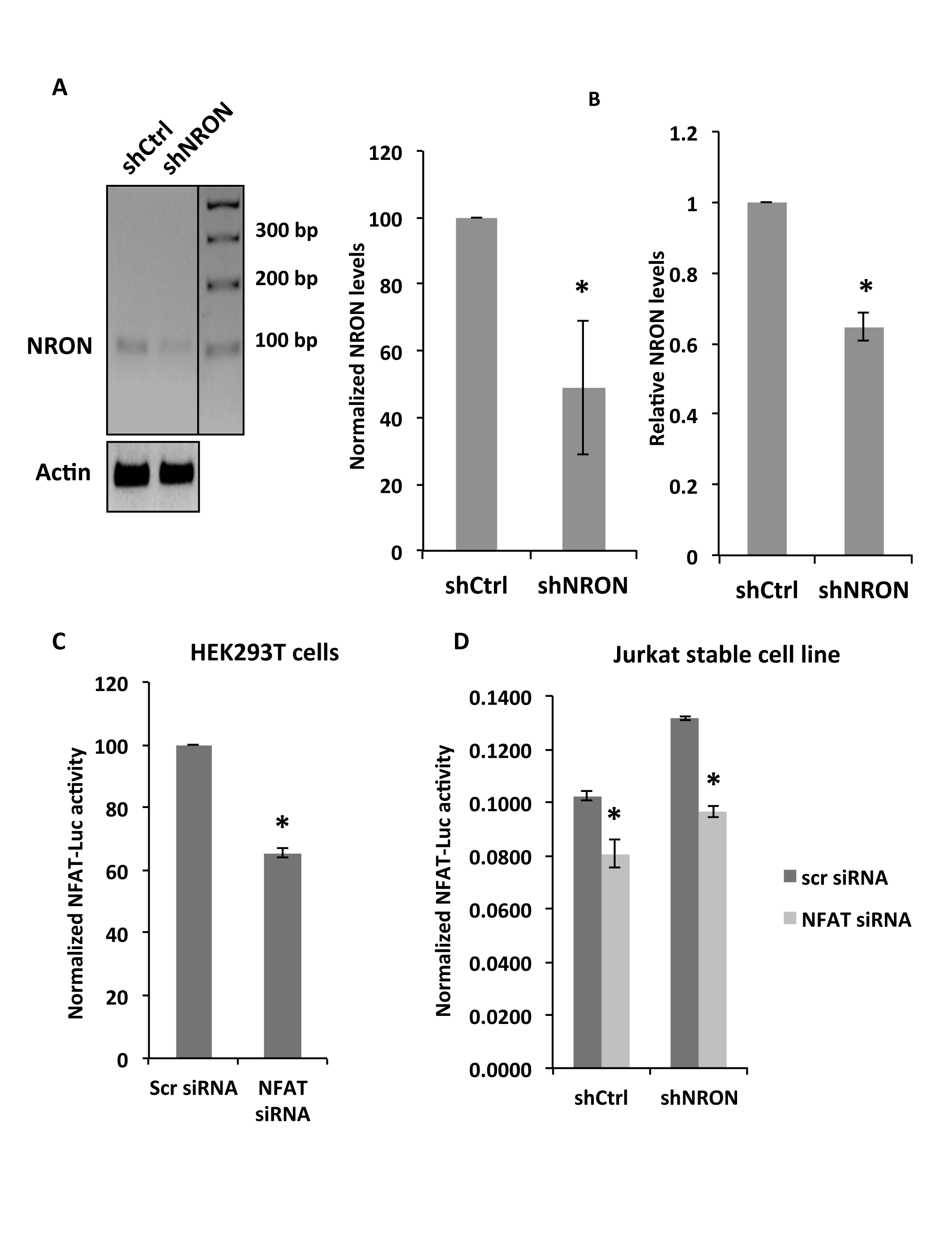
**

**Figure S3: Decreased NRON expression in NRON knockdown cells**. (A) Semi-quantitative RT-PCR for NRON levels in control and NRON knockdown cells. The cropped gels show NRON and Actin RNA levels run on a 2% agarose gel. Estimations were made at 48 hr after plating the cells. (B) Quantitative RT-PCR for NRON levels in control and NRON knockdown cells at 48 hr after plating. Efficacy of siRNA-mediated NFAT knockdown in (C) HEK293T cells and (D) Jurkat NRON knockdown and control cell lines as described in Methods. The NFAT activity was determined 48 hr after co-transfection of the pNFAT-luc reporter plasmid and plasmid pRLTK (to control for transfection efficiency). Results from three separate experiments are shown as mean values +SD; * p<0.05.


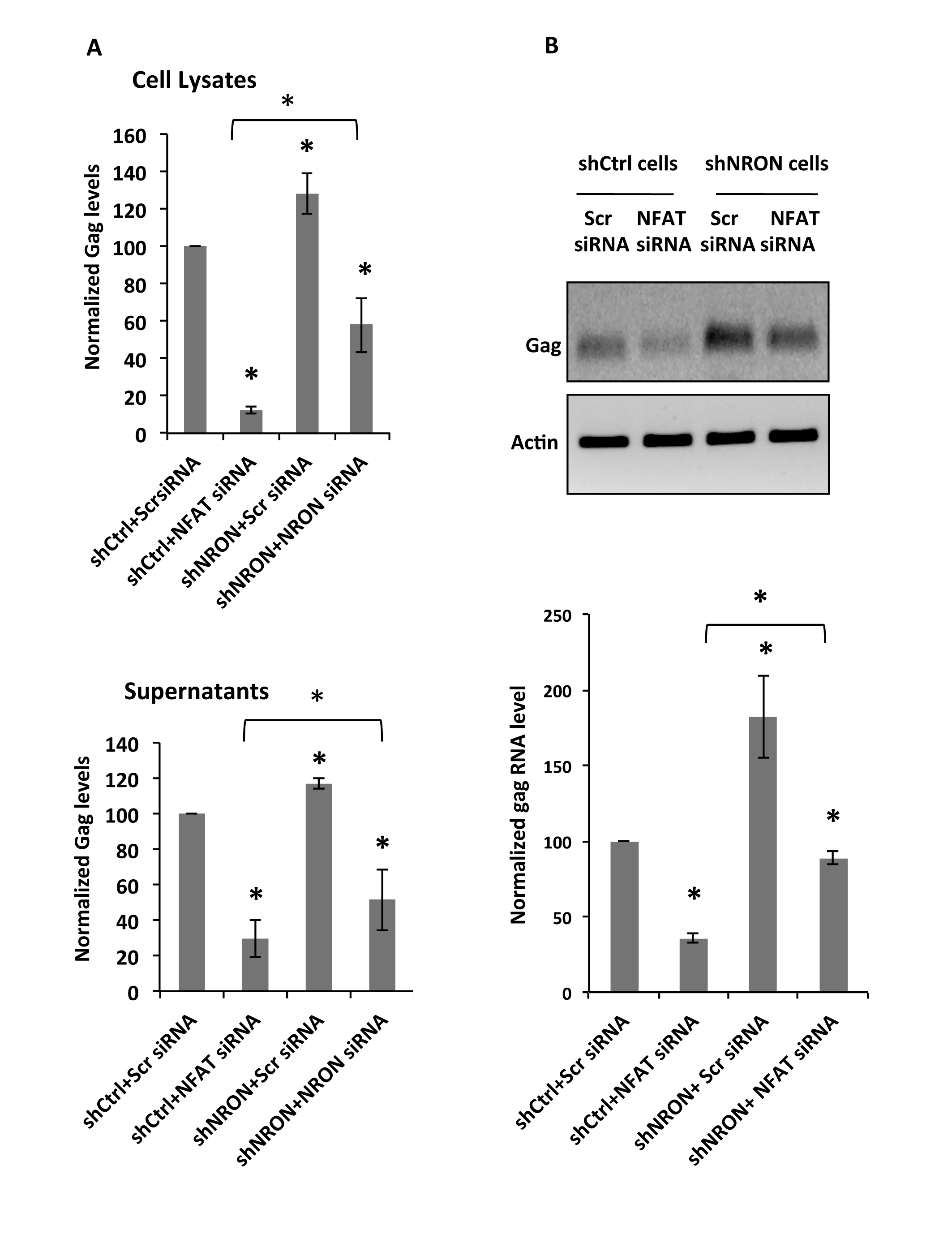


**Figure S4:** (A) Densitometric quantitation of Figure 4E. Individual values for lysates (p55 and p24) and supernatants (p24) were taken from each lane and presented as a percentage, taking the values in lane 1 as 100%. (B) Gag RNA expression levels were checked by semi- quantitative RT-PCR. Cropped gel image shows Gag and Actin levels in control and NRON knockdown cell lines, semi-quantitative RT-PCR products were run in 2% agarose gel in same experimental condition. For densitometric quantitation individual values for PCR products were taken for each lane and presented as a percentage, taking the values in lane 1 as 100%. Results from three separate experiments are shown as mean values +SD; * p<0.05.


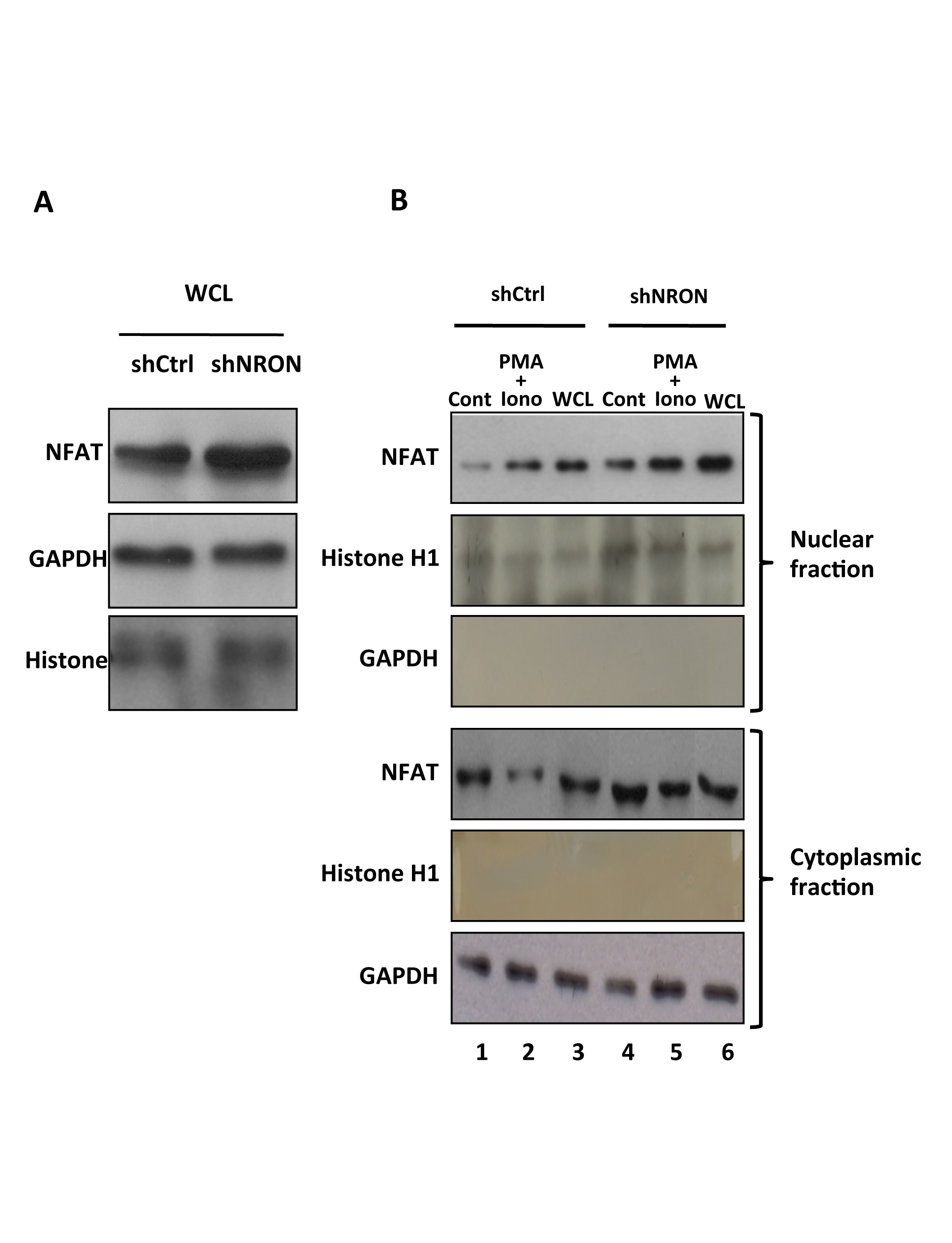


**Figure S5:** **NRON knockdown cause elevated level of nuclear NFAT protein**. (A) More NFAT protein was observed in the whole cell lysates of NRON knockdown cells compared to control knockdown cells, at 48 hr after plating. GAPDH and Histone served as loading controls. (B) Control or NRON knockdown Jurkat cells were kept as such (Cont) or stimulated with PMA/Ionomycin for 3 hr after 48 hr of plating. The cells were fractionated into nuclear and cytoplasmic fractions as described in Methods and western blotted for NFAT protein levels. GAPDH and Histone H1 (HH1) served as cytoplasmic and nuclear markers, respectively. The whole cell lysate (WCL) of unstimulated cells was also run for comparison.


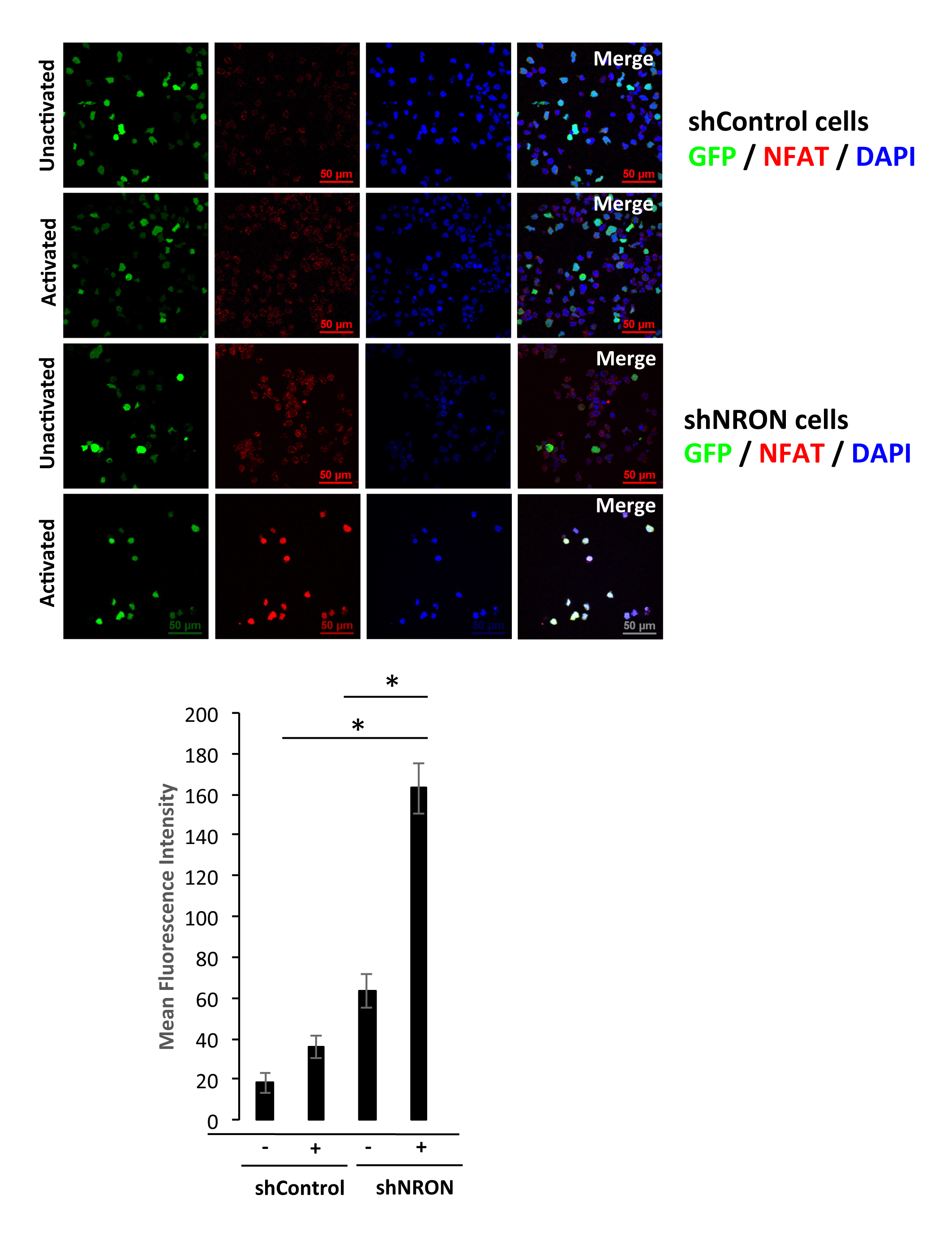


**Figure S6: Nuclear translocation of NFAT protein in NRON knockdown cells.** Control or NRON knockdown Jurkat cells (green) were stained for NFAT (red) and nuclei (DAPI, cyan) with or without activation with PMA/Ionomycin. The fluorescent images are shown. The graph on the right shows Mean fluorescence Intensity (MFI) of the two cell lines without (-) or with (+) PMA/Ionomycin activation. Data is representative of MFI of 35 cells from three different replicates; (* p<0.001).
